# Postharvest Management Practices of Grains in the Eastern Region of Kenya

**DOI:** 10.5539/jas.v11n3p33

**Published:** 2019-02-15

**Authors:** Anastasia W. Njoroge, Ibrahim Baoua, Dieudonne Baributsa

**Affiliations:** 1 Department of Entomology, Purdue University, Indiana, USA; 2 Department of Entomology, Université de Maradi, Maradi, Niger

**Keywords:** storage, drying, grain, hermetic methods, semi-arid

## Abstract

Cereals and legumes play a major role in the production systems and diets of farmers in the semi-arid eastern region of Kenya. Efficient postharvest management can tremendously contribute to food security in these regions. A study was carried out in three counties in eastern Kenya to assess pre and postharvest management practices among farmers. Data was collected using semi-structured questionnaires designed and administered using Kobo Toolbox via android tablets. Results showed that farmers cultivated three main crops: maize (98%), beans 66%), and pigeon peas (28%). The most saved seed crops were beans (80%) and pigeon peas (50%). Majority of the farmers (80%) experienced pre-drying losses due to insects (48%), rodents (40%) and birds (39%). Farmers stored grain for consumption (80%) and for sale (19%). About 48% of farmers stored the grain for more than 9 months. Challenges during grain storage were insects (57%) and rodents (43%). Primary methods of grain preservation included hermetic methods (61%) followed by insecticides (33%). While progress is being made in addressing storage challenges, there still a need to continue building awareness about improved storage technologies and find solutions for pest infestations in the field and drying after harvest.

## 1. Introduction

Cereals and legumes are the most important food staples in Sub-Saharan Africa (Abate et al., 2012; Macauley & Ramadjita, 2015). They are often grown alone or in intercropping systems (Van Duivenbooden et al., 2000). Up to 90% of agricultural production in East Africa is dominated by smallholder farmers and about 75% of Kenyans owe their livelihood to agriculture (FARA, 2006; Stathers et al., 2013; Wiggins & Keats, 2013). Smallholder agriculture is often faced with low productivity due to inefficient production systems.

Most grain in Kenya is produced in the Rift Valley, Western, Nyanza, Central and Eastern regions (Rao et al., 2015). Eastern Kenya produces cereal and legume crops including maize (*Zea mays* L.), beans (*Phaseolus vulgaris* L.), green grams (*Vigna radiata* L.), pigeon peas (*Cajanus cajan* L.), millets (*Pennisetum* spps), sorghum (*Sorghum bicolor* L.), cowpea (*Vigna uncguiculata* L.) and dolichos lablab (*Lablab purpureus* L.) to ensure self-sufficiency in the face of climate change (Mergeai et al., 2001; Kimiti et al., 2009; Muhammad et al., 2010; County et al., 2016). The demand for staple crops such as maize is expected to continue to grow despite the diversification of Kenyan diets. Like all farmers in Sub-Saharan Africa (SSA), farmers in Kenya face many challenges during crop production including widespread changes in rainfall and temperature patterns. Changes in weather patterns threaten agricultural production and increase the vulnerability of people who depend on agriculture for their livelihoods (Speranza et al., 2008; Tabu et al., 2013).

In Kenya, grain production and national consumption patterns do not tally. This is common, especially for maize, as it does not meet national demand (Gitonga et al., 2013). Often, the deficit is met by imports or food aid. In addition, demand for maize in the processing of animal feeds is also expected to increase due to a recent major investment in the subsector (Gitonga, 2017). Annually, Kenya produces up to 3.6 million MT of maize while it imports up to 1.5 million MT in some years to meet its food demand (FAO, 2014). With postharvest losses estimated at 30% (Mutambuki & Ngatia, 2006), Kenya loses about half a million MT each year. Efforts to mitigate postharvest losses have the potential to reduce or eliminate maize imports into Kenya. Improved postharvest management can increase food supply without the need to use additional resources such as land, water, seed and fertilizer.

Crop losses occur before and after harvest and include inadequate drying, inefficient storage facilities, and lack of appropriate technologies. Drying is done in the field and at home while storage is done in or near the house using different containers and technologies. Storage is often plagued by a myriad of problems such as insects, mold, birds, rodents and animals. Therefore, it is important to understand farmers’ pre and postharvest management practices of key important cereal and legume grains in the semi-arid region of Eastern Kenya to improve food security and income of farm households.

## 2. Methodology

### 2.1 Study Area

**Figure 1:**
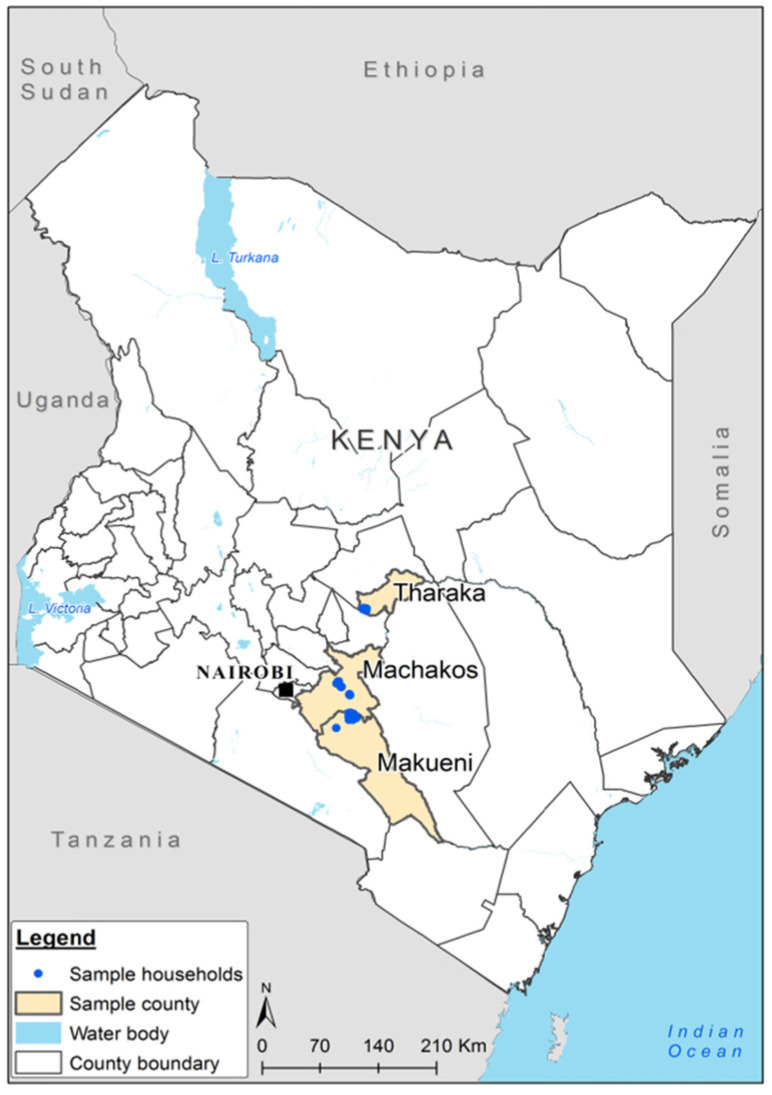
Map of Kenya showing the study sites (in blue dots) in three counties in Eastern Kenya *Source*: Data collected during the study and mapped using QGIS software.

This study was conducted in three counties (Tharaka Nithi, Makueni and Machakos) in the Eastern region of Kenya (Fig. 1). The survey was implemented from October 9 to November 3, 2017 targeting farmers who had harvested and stored grain from the previous major (long rainy) season. The Eastern region is mainly semi-arid with low and erratic rainfall that results in periods of severe drought. This area is prone to government hunger and poverty interventions. The region has bimodal rainfall characterized by a long rainy season from March– May and short rainy season between September and December (Jaetzold et al., 2006).

### 2.2 Sampling, Data Collection and Analysis

We purposively selected 50 villages in the three counties (16 villages in Tharaka-Nithi, 17 villages in Makueni, and 17 villages in Machakos). The number of villages per country was determined based on cereal and legume production. We targeted to interview 13 farmers randomly selected in each village. We were able to survey a total of 613 farmers (94.3% of our goal). The selected farmers were interviewed using a semi-structured questionnaire with open and close-ended questions. Questions included qualitative and quantitative data focused on the socio-economic characteristics of the respondents; major cereal and legume crops produced, the quantity produced; seed sources and storage, grain drying before and after harvest, storage practices; farmers’ knowledge of the causes of postharvest losses and loss prevention measures. The questionnaire was uploaded to Kobo Toolbox, deployed and administered via handheld devices (android tablets). Five enumerators collected data and received consent from individual farmers before each interview. Data collected were coded and analyzed using the SPSS 24.0 (IBM Corp., 2016, New York, NY, United States). Cross-tabulation tables were constructed, and descriptive analyses done to summarize the data.

## 3. Results

### 3.1 Characteristics of the Respondents

Table 1 summarizes the characteristics of the respondents. Among the respondents: 64% were female, 87% were married, 49% were above 50 years of age, and 57% had more than a high school education. The average household size was five members. About 82% of the respondents had 10 years or more of experience in their main activity. Most respondents (85%) were smallholder farmers owning less than 5 acres of land.

**Table 1:** Characteristics of respondents and their socio-economic attributes (%) in the three counties surveyed in Eastern Kenya

Variable	% Respondents			Overall (n=613)
	Tharaka-Nithi (n=170)	Makueni (n=216)	Machakos (n=227)	
*Gender*				
Female	15.3	23.2	25.9	64.4
Male	12.4	12.1	11.1	35.6
*Age*				
18-30	2.4	2.9	3.3	8.6
31-40	5.9	5.7	6.9	18.4
41-50	7.0	8.5	8.5	24.0
over 50	12.4	18.1	18.4	48.9
*Marital status*				
Single	1.8	1.3	1.0	4.1
Married	24.0	30.5	32.6	87.1
Widowed	1.5	3.3	2.9	7.7
Separated/divorced	0.5	0.2	0.5	1.1
*Education level*				
None	0.3	1.0	0.5	1.8
Basic Literacy	2.1	1.6	1.8	5.5
Primary School	9.5	12.2	13.9	35.6
High School	10.6	12.2	13.5	36.4
Tertiary/University	5.3	8.1	7.3	20.7
*Main activity*				
Full-time employee	8.2	3.7	4.8	5.4
Business	7.1	10.2	11.5	9.8
Agriculture	82.4	86.1	82.4	83.7
*Years in activity*				
Less than 5	75.9	87.5	81.1	81.9
5-10	17.1	8.8	13.2	12.7
More than 10	7.1	3.7	5.7	5.4
*Land size (acres)*				
0-1	9.3	6.2	5.7	21.2
1-5	16.2	21.4	26.3	63.8
5-10	1.5	4.9	3.8	10.1
More than 10	0.8	2.8	1.3	4.9

### 3.2 Main Cereal and Legume Crops Cultivated

Table 2 provides details of the first three main crops grown by respondents. Overall across the three counties, the main food crops were maize (98%), common beans (66%) and pigeon peas (28%). Respondents in Tharaka-Nithi county predominantly farmed two main crops (cereal and a legume), while those in Makueni and Machakos counties grew three main crops. Majority of the respondents (69%) said the main reason for growing cereal and legume grains was for both sale and consumption.

**Table 2:** Main cereal and legume crops cultivated by farmers in three counties in Eastern Kenya

Crop	% Respondents						
	Maize	Beans	Pigeon pea	Cowpea	Green gram	None
First main crop	98.0	-	-	-	-	-
Second main crop	-	66.7	20.7	6.0	3.4	-
Third main crop	-	18.4	28.4	11.4	6.9	34.8

### 3.3 Drying Practices

Table 3 summarizes farmers drying practices. Farmers pre-dried their crops in the field. Eighty percent (80%) of farmers experienced postharvest losses during the pre-drying period. The main causes for these losses were insects (48%) followed by rodents (40%) and birds (39%). All respondents reported having challenges during drying of grain prior to storage. A little over half of farmers (55%) mentioned rain as the main challenge during the drying period. Majority of the respondents (98%) mentioned drying in the sun as the most commonly used method for reducing grain moisture content to acceptable levels before storage. About two third of the respondents (65.4%) dried on the ground while 21.7% used a mat/tarpaulin for drying.

**Table 3:** Summary of pre-drying losses, grain drying practices and challenges to farmers in three counties in Eastern Kenya

Variable	% Respondents
*Pre-drying losses*	
Mold	28.7
Animals	27.4
Theft	12.9
Rodents	39.2
Birds	40.1
Insects	48.0
*Drying systems*	
Field	21.4
Shade	2.3
Sun	97.7
Smoking	3.3
House	3.9
*Drying surfaces*	
Ground	65.4
Mat/Tarpauline	21.7
Others	10.6
*Drying challenges*	
Rain	53.5
Drying space	17.9
Contamination	13.7

### 3.4 Storage Practices

Tables 4, 5 and 6 provide summaries of crop storage practices. Maize was the most produced and stored crop followed by common beans (Table 4). Across the three counties, the main reason to store was home consumption (80%) followed by income security (19%). Most farmers (73.9%) stored for 6 months or more (Table 5). The primary method of grain protection was hermetic methods (61%) followed by chemical pesticides (33%). A little over half of the farmers (55.6%) store their harvested grain in a room in the house. Insect damage was the biggest challenge during grain storage (57%) followed by rodent damage (43%) (Table 6). Most farmers dealt with these challenges by turning to new storage technologies (46%). A good number (54%) did not use chemicals. The main reason was because chemicals are toxic/harmful to their health (56%). Majority of the farmers saved grains as seed from the previous harvest for replanting in the next season (Table 7). But the quantity saved varied from one crop to another—80% for common beans, 50% for pigeon peas and 20% for maize. Though farmers saved seed, 86% also mentioned buying seed from agrodealers’ shops.

**Table 4:** Summary of first three major stored crops grown, and quantity produced and stored by farmers in three counties in Eastern Kenya

3 major crops	Crop produced	Quantity produced (bags^*^)			Quantity stored (bags^*^)		
		0-5	6-10	>10	0-5	6-10	>10
Maize^**^	98.0	35.4	27.9	36.7	60.0	23.5	16.5
Beans	66.7	85.5	10.3	4.3	93.5	4.3	2.2
Pigeon pea	28.4	97.4	1.6	1.0	98.5	1.2	0.3

*Note.* * Each bag has the capacity to store 90 kg of grain. **All data in the table are expressed as percent respondents.

**Table 5:** Grain storage practices—duration of storage, method of grain protection and location of storage—by farmers in three counties in Eastern Kenya

Variable	% Respondents
*Duration of storage*	
Less than 3 months	6.7
3-6 months	19.1
6-9 months	25.8
More than 9 months	48.1
*Method of preservation*	
Do nothing	4.4
Chemical pesticides	32.8
Natural products	0.8
Hermetic technologies	61.0
Others	0.5
*Location of storage*	
Room in house	55.6
Farm store	13.9
Improved granaries	25.3
Traditional granaries	12.4

**Table 6:** Grain storage practices—challenges during storage, methods of dealing with storage challenges, and reasons for not using chemicals—by farmers in three counties in Eastern Kenya

Variable	% Respondents
*Challenges during storage*	
Insect damage	56.8
Rodent damage	43.1
Ineffective insecticides	17.8
Decay/mold	7.2
Theft	2.6
*Method of dealing with storage challenges*	
Use new technologies	46.3
Apply chemicals	38.5
Feed livestock	14.0
Sell at harvest	17.6
Consume	11.9
*Reasons for not using chemicals*	
No insect attacks	9.6
Expensive	2.9
Not effective	9.0
Toxic	11.4
Others	16.9
Don’t use chemicals	50.2

**Table 7:** Percent of farmers who saved seed and quantity of seed saved in three counties in Eastern Kenya

Variable	% Respondents		
	Beans	Pigeon pea	Maize
Farmers who saved seed	80	50	20
Quantity saved by farmers			
Less than 10 kg	64	80	92
10- 30 kg	21	12	5
More than 30 kg	15	8.5	3.2

## 4. Discussion

### 4.1 Characteristics of the Respondents

About half of the farmers were an aging demographic, above 50 years. Older farmers are usually viewed least productive because most field work require physical effort. In addition, this may be a hindrance to adoption of new technologies which has a negative impact on food security. This is a universal trend caused by youth migration to urban centers among other factors. Policy-makers need to develop programs that incentivize the youth to be involved in agriculture. The average household size was five members which is similar to the Kenyan national average of between 4.9 to 5.7 people (Munene, 2003). Bigger households mean more family-labor and more incentives to produce more grain and store more for family consumption. The respondents had farming experience and had attained at least high school education which may positively influences their ability to make sound postharvest management decisions. It was apparent during the study that women played an important role in postharvest management in Eastern Kenya. Similar findings have been reported in West and Central Africa (Ibro et al., 2014; Moussa et al., 2014).

### 4.2 Main Cereal and Legume Crops Cultivated

The three major crops grown across the three counties were maize, beans and pigeon pea. Most farmers grew at least two major crops including a cereal and a legume. This agrees with the report that farmers in semi-arid areas of eastern Kenya grow two major and one minor grain crops (Wambugu & Muthamia, 2009). Farmers cultivated cereals and legumes for food, feed and sale. Almost all farmers grew maize and most of it was hybrid-maize. Most farmers who grew maize sourced their seed from agrodealers as they saved little of their harvested grain. We think that the saved maize seeds are local varieties that are grown by farmers for family use or niche markets. The adoption of hybrid maize in Kenya has been increasing over the last few years and is high compared to other countries in the region (Schroeder et al., 2013). This is in part due to efforts by seed companies to produce hybrids that are adapted to local weather such as the semi-arid conditions. Studies have shown low hybrid seed adoption rates are common across developing economies worldwide, especially in Sub-Saharan Africa where it is approximately 40 percent (Smale et al., 2011). In contrast to maize, farmers saved their own legume seeds for replanting the next season. Saved legume seeds can still provide good yield if proper selection of planting material is done, since they are self-pollinating. Providing farmers with relevant information and knowledge about seed sourcing (whether saved or purchased) is critical as it affects their production. Planting good seed ensures good production.

### 4.3 Drying and Storage Practices

There was little variation in postharvest practices among the three study sites. Ideally, rainfall in these regions is bimodal with the long rains occurring from March to May and short rains from October to December. However, all these counties have been experiencing an overall decrease in precipitation and increase in temperatures, with negative impact on crop yields (Ojwang’ et al., 2010; Gichangi et al., 2015). Farmers had several challenges during preharvest, the major ones being field infestations by insect, birds and rodents. There are limited solutions to deal with these preharvest issues. This may explain why fewer farmers were pre-drying their crops in the field. Drying has always been a challenge among smallholder farmers. This is often the case in areas where there are bimodal rainfalls such as Eastern Kenya. Sometimes the harvest season for one crop overlaps with the next planting, which is usually dictated by rainfall. This creates drying challenges for the mature crop to be harvested. Rain as a major challenge during drying was mentioned by more than half of respondents. Technologies are being developed to address drying challenges including the EasyDry M500 which is a mobile portable maize dryer targeted at servicing smallholder farmers (Walker & Davies, 2017). With two thirds of farmers drying their crops on the ground, this may explain the high level of aflatoxin contamination in Kenya (Mwihia et al., 2008). The introduction of proper technologies will address issues of drying grain during rainy season including mold development and aflatoxin accumulation.

Farmers had a good understanding of storage and its implications for food security. Studies have shown the importance of storage in enhancing food security in semi-arid areas (Nduku et al., 2013). Regardless of quantity produced, majority of farmers stored at least two bags for home consumption. Smallholder farmers who owned less than two acres of land, especially in Tharaka-Nithi county, stored grain for home consumption. Though land sizes were a bit larger in Machakos and Makueni counties, adverse effect of changing weather patterns reduced their productivity. About four-fifths of the respondents stored their grain for more than 6 months. Similar trends have been observed in Tanzania where farmers stored for six or more months to secure food and get higher prices for their grain. Farmers need to store for at least six months to achieve the highest prices due to price seasonality of grain in African markets (Jones et al., 2011). Some farmers even stored for 12 months or longer. The main reason for longer storage was to build a buffer stock due to unpredictable weather patterns that may result in poor harvest in the following seasons. During the study, we even found some farmers who were storing cereal and legume grains for 24 and 36 months. Long storage was made possible with access to better storage technologies such as hermetic bags including the Purdue Improved Crop Storage (PICS), GrainPro, and AgroZ bags (Foy & Wafula, 2016). Thanks to several efforts to reduce postharvest losses, these hermetic bags have been promoted in several counties by the Kenya Agriculture Value Chains (KAVES) Program and other projects (Nash et al., 2017). Cereal and legume crops can be stored or preserved for a very long time without major pre-processing (Appert, 1987). Better storage also helps preserve seed and avert agents of qualitative and quantitative grain loss like insects, rodents and mycotoxins (Adetunji, 2007). Qualitative loss through aflatoxin contamination of maize has been widely reported in Kenya, and especially in Machakos and Makueni counties (Kang’ethe, 2011; Maina et al., 2016). With hermetic bags, damage by pests and contamination by aflatoxins are mitigated (Njoroge et al., 2014; Maina et al., 2016; Williams et al., 2014).

### 4.4 Storage Challenges

The major challenges during storage were insects and rodents. Farmers reported using new storage technologies and insecticides to deal with these pest challenges. Farmers have relied on pesticides for grain preservation, but they have been shifting towards hermetic storage in recent years. Concerns with pesticides stem from food poisoning due to overuse and misuse of insecticides as well as their ineffectiveness linked to insect resistance (Nicolopoulou-Stamati et al., 2016). The availability of cost-effective storage options has increased farmers’ willingness to invest in safe storage technologies. The introduction of various hermetic technologies in Kenya since 2013 followed by aggressive marketing and advertising has helped increase the adoption of these technologies. Studies have shown that hermetic storage for 6 months is more profitable than the use of chemicals (Foy & Wafula, 2016). A study implemented in Uganda showed an increase in farmers’ willingness to invest in hybrid maize varieties after accessing better storage technologies (Omotilewa et al., 2018). Farmers who had used hermetic bags for two years were 10 percent more likely to plant hybrid maize (high yielding but more susceptible to insect during storage) the following season compared to those who were using other storage methods.

Overall, farmers have a greater understanding of postharvest practices in the three counties in Eastern Kenya. Maize and common beans are the most important crops grown by farmers in these areas. Postharvest challenges linked to drying and storage such as aflatoxins and insects are serious challenges. Farmers are increasingly accessing new technologies and solutions to deal with these issues. Maize being such a staple crop grown by all farmers, there is a need to improve and promote pre-harvest and postharvest management practices to increase the use of new technologies such as improved seed, dryers, and hermetic storages devices.
